# Programmes Addressed to Informal Caregivers’ Needs: A Systematic Literature Review

**DOI:** 10.3390/geriatrics9030071

**Published:** 2024-05-30

**Authors:** Laurência Gemito, Elisabete Alves, José Moreira, Maria Fátima Marques, Ermelinda Caldeira, Rogério Ferreira, Isabel Bico, Lara Pinho, César Fonseca, Luís Sousa, Manuel Lopes

**Affiliations:** 1 São João de Deus School of Nursing, University of Évora, 7000-811 Évora, Portugal; mlpg@uevora.pt (L.G.); elisabete.alves@uevora.pt (E.A.); jose.moreira@uevora.pt (J.M.); mfm@uevora.pt (M.F.M.); ecaldeira@uevora.pt (E.C.); isabelbico@uevora.pt (I.B.); cfonseca@uevora.pt (C.F.); mjl@uevora.pt (M.L.); 2 Comprehensive Health Research Centre (CHRC), 7000-811 Évora, Portugal; ferrinho.ferreira@ipbeja.pt (R.F.);; 3 School of Health of Beja, Polytechnic Institute of Beja, 7800-111 Beja, Portugal; 4 School of Health Atlântica (ESSATLA), Atlântica University, 2730-036 Barcarena, Portugal

**Keywords:** ageing, health outcomes, informal caregivers, needs assessment, systematic review

## Abstract

Background: Addressing informal caregivers’ needs is essential for ensuring quality healthcare and promoting citizen-centred care. This systematic review assessed current knowledge about programmes aimed at meeting the needs of informal caregivers of adults who are dependent on others for daily life activities. Methods: Following the PRISMA guidelines, the electronic databases EBSCOhost Research Platform, MEDLINE, CINAHL, Scopus, Web of Science and The Virtual Health Library were searched for randomized experimental studies published between 2012 and 2022 that implemented programmes addressing informal caregivers’ needs to improve their experiences, health, and well-being. Quality was assessed using the standardized critical evaluation tools from the Joanna Briggs Institute. Two independent investigators performed the eligibility assessment and data extraction. Quantitative data on the effectiveness of interventions were collected, and the content of each intervention was synthesized and aggregated into categories, through narrative synthesis. Results: The majority of the included studies (n = 16) were conducted in European countries and implemented a structured intervention programme compared to the provision of usual care. The studies were of fair to high methodological quality, with a higher risk of bias related to blinding. The results supported the achievement of favourable health outcomes among informal caregivers, namely improvements in mental health (n = 3) and quality of life (n = 3) and a decrease in psychological symptomatology (n = 5) and burden (n = 3). None of the interventions reported adverse outcomes; however, five studies did not describe significant differences in the outcomes assessed after the implementation of the programmes. Interventions focusing on training and educating caregivers (n = 14) and cognitive–behavioural strategies (n = 7) were the most common, while programmes focusing on emotional and psychological support as a resource to improve caregivers’ psychological outcomes were scarce. Conclusions: This systematic review adds to the growing body of evidence and insight showing that programmes that address informal caregivers’ needs seem to contribute to better physical and psychological health outcomes through the promotion of caregivers’ educational support and the implementation of cognitive–behavioural strategies. Future research should implement methodologically robust cross-country programmes tailored to informal caregivers’ physical, emotional, psychosocial, societal, and educational needs throughout the care trajectory.

## 1. Introduction

Globally, the number of older people (i.e., aged 65 and above) has increased, reaching 761 million in 2021 [1]. This number is projected to grow further, with the proportion of older persons expected to increase from 20% in 2023 to 28% in 2050 [2,3]. In Europe, the population of older people is also expected to increase and is estimated to reach 129.8 million in 2050 [4]. In 2020, the old-age dependency ratio was 34.8%; that is, there were slightly fewer than three people of working age for every older person. Population projections for 2050 suggest that the old-age dependency ratio in the EU-27 could reach 56.7%, which represents less than two people of working age for every older person [4,5].

Increased life expectancy has been associated with increased multimorbidity and frailty [6]. This has led to an increase in the number of older people with health and social assistance needs [7], despite the decline in life expectancy observed in 2020 in high-income countries due to COVID-19 [8]. In fact, in recent decades, the health-adjusted dependency ratio has revealed a greater association with growth in health expenditure than the dependency ratio of older persons [9].

To lessen the pressure on formal care systems, policies have emerged in several European countries to encourage older people to live longer in their own homes and receive home-based health care [6]. Family members are the main source of support for people with chronic illnesses and disabilities [10] and for frail older adults with multimorbidity [11]. An informal caregiver is the person who makes the most time available to monitor and satisfy the needs of people who depend on others for basic and instrumental daily life activities. This role is often performed by a close family member (spouses, children) who, in most cases, is unpaid [12].

A meta-analysis revealed that 62% of informal caregivers are female, with a mean age of 53 years. Most caregivers (68%) take care of their spouse or partner for 41 h a week and for at least 7 years on average [13]. The loss of independence in carrying out activities of daily living for family members and the consequent need for care cause informal caregivers to change their routines, lifestyles, and family dynamics, leading to negative consequences for their physical and mental health [12,14].

In this regard, many European countries have made significant reforms to long-term care policies and systems to provide greater responsibility and support for informal caregivers based on their status [15]. However, there are several aspects that need to be optimized with regard to informal care to improve caregivers’ experiences, health, and well-being. It is therefore extremely important to know caregivers’ needs and expectations. At the same time, the negative and exhausting consequences that informal caregivers are subject to, such as the high level of overload, the effects on physical condition, mental health, stress, and the reorganization of dynamics at the family level [16], should also be considered.

Given both the expectations of informal caregivers and the consequences that arise from their situation, previous studies have structured their main needs into four domains: (1) information on issues related to mobility and the prevention of falls, illness, and death; (2) social, financial, and health support; (3) balance between family and professional life; and (4) social recognition [15,17]. Overall, the literature indicates that to improve the quality of health care through positive results and to promote care centred on older people and their families, it is essential to account for the different needs of informal caregivers [18]. These needs may vary depending on the type and stage of disease [19,20]. The literature shows that psychosocial strain and burden among caregivers of people with cognitive disorders due to Alzheimer’s disease may evolve with the progression of the disease and the transition from dementia [21,22].

In this context, the following research question was formulated based on the PICO(D) mnemonic [23]: What are the health gains for the person being cared for, the informal caregiver, or the health system (O) resulting from the implementation of programmes that address informal caregivers’ needs to improve their experiences, health, and well-being (I), compared to usual care (C) among informal caregivers of adults with dependence for daily life activities (P)? Through a systematic literature review, we identify the current state of knowledge on programmes that address the needs of informal caregivers.

## 2. Materials and Methods

### 2.1. Study Design, Protocol, and Registration

For this systematic review, a protocol was registered on the PROSPERO platform under the number CRD42021241297, prepared, and published [17]. Randomized controlled trial studies were considered to provide greater specification and a better understanding of the different interventions with informal caregivers. Given the heterogeneity of the methodological designs, explicit information in the studies was explored through a narrative and thematic synthesis approach to combine the different results.

This systematic review followed the Preferred Reporting Items for Systematic Reviews and Meta-Analysis (PRISMA) 2020 guidelines [24].

### 2.2. Eligibility Criteria

To define the inclusion criteria, we used the PICO(D) framework [25]. Studies that had the defined characteristics for each element of the PICO(D) framework and that reported at least some types of main outcome were included (Table 1). Eligible publications were restricted to those published in English, Spanish, Portuguese, French, or Italian in the last 12 years (January 2012 to May 2024) for which the full text was available. All papers selected for full-text analysis were written in either English or Portuguese, eliminating the need for translation.

### 2.3. Data Source and Search Strategy

#### 2.3.1. Data Sources

To identify the most relevant studies on this subject, bibliographical research was conducted in the following databases: EBSCOhost Research Platform (CINAHL^®^ Plus with Full Text; Nursing & Allied Health Collection; Cochrane Plus Collection, including Cochrane Central Register of Controlled Trials; Cochrane Database of Systematic Reviews (CDSR) and Database of Abstracts of Reviews of Effects (DARE); MedicLatina; MEDLINE^®^, including International Nursing Index); PubMed via MEDLINE; CINAHL; Scopus; Web of Science; The Virtual Health Library (VHL); and Open Gray and Gray Literature Report. The search strategy for each database has been previously described [17].

#### 2.3.2. Search Strategy

The research was conducted based on terms recommended by the MESH: ((Caregiver) OR (Care Giver*) OR (Caregiver*, Family) OR (Caregiver*, Spouse)) AND ((Disabled Persons) OR (Elderly Dependent) OR (Aged) OR (dementia) OR (Alzheimer’s) OR (Disability) AND ((Care, Non-Professional Home) OR (Nonprofessional Home Care) OR (Old Age Assistance) OR (Patient-Centered Care) OR (Patient-Centered Nursing) OR (Patient-Focused Care) OR (needs assessment) OR (care management) OR (health care) OR (psychosocial care) OR (community) OR (Community-Based Distribution).

### 2.4. Identification and Selection of Studies

The identification and selection of studies was conducted according to the proposed objective of identifying the current state of knowledge about programmes that aimed to meet the needs of informal caregivers. This selection was structured in accordance with the PRISMA statement [24] after reviewing the different titles and abstracts of the various studies, excluding those that did not meet the defined criteria.

In addition to the inclusion criteria, the articles were examined by two independent reviewers. Discrepancies between the two reviewers were resolved by discussion of the article or by the intervention of a third reviewer. All reviewers had backgrounds in health and social sciences, as well as work experience in ageing processes, caregiver health and well-being, and community interventions. This ensured that they were qualified to identify and select studies for this systematic review.

### 2.5. Quality Appraisal

Randomized controlled trials selected from the retrieved studies were examined by two independent reviewers for methodological validation using standardized critical evaluation tools from the Joanna Briggs Institute (JBI) Review [26] and Statistical Evaluation of Meta-Analysis Instrument (JBI-MAStARI) for inclusion in the review. Evidence levels were classified according to the JBI [27]. Discrepancies between the reviewers’ assessments were resolved through discussion. When there was no consensus, a third reviewer was included in the discussion.

The JBI checklist score was as follows: “Yes” with 1 point, and “No” and “Unclear” with 0 points. The sum of the points was classified from 70% of the items present based on the recommendations of Camp and Legge [28]. Thus, a score of 70–79% of the checklist criteria was classified as medium quality, a score of 80–90% was considered high quality, and a score greater than 90% of the criteria was classified as excellent quality.

### 2.6. Strategy for Data Extraction and Data Synthesis

#### 2.6.1. Data Extraction

The strategy for data extraction, in an initial phase, was conducted in a summarized and descriptive way by analyzing each article, considering information on the authors and year of publication, the country where the study was conducted, the period of data collection, the participants and sample, the intervention implemented, the outcomes assessed, the instruments used, and the main results of the interventions. Data on the specific content of each intervention were also retrieved. The extraction of data from these selected articles was independently performed by two researchers. In situations where there was a discrepancy in the assessments of the two researchers, this discrepancy was resolved by the intervention of a third reviewer. Quantitative data on outcomes whose association with the interventions was statistically significant were collected, and the directions of the associations were recorded.

#### 2.6.2. Data Synthesis

Following the PRISMA guidelines [24], key summary statistics on the effectiveness of interventions were collected whenever available (*p*-values, effect sizes, confidence intervals, etc.), and the directions of the associations were registered. Subsequently, through narrative synthesis [29], it was possible to synthesize and aggregate the content of each intervention into categories based on the similarity of meaning. This synthesis of results obtained from different studies through quantitative data and narrative text analysis allowed the identification of the different types and dimensions of interventions aimed at meeting the needs of informal caregivers.

## 3. Results

### 3.1. Description of Study Selection

**Figure 1:**
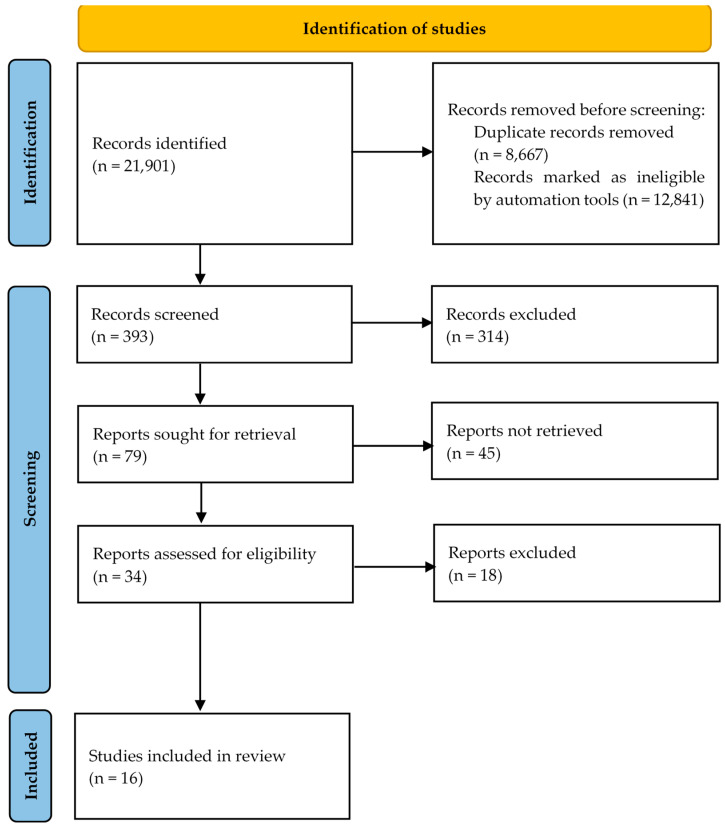
PRISMA 2020 systematic review flowchart of the selection of studies.

The search results and the screening process of this systematic review are presented in detail in the flowchart in Figure 1 [24]. The search resulted in a total of 21,901 articles. After duplicate results (n = 8667) and records marked as ineligible by automation tools (n = 12,841) were eliminated, 393 articles were examined. Of these, 314 articles were eliminated because they did not meet the inclusion criteria. Of the 79 articles selected for full-text analysis, 45 were not retrieved. Among the remaining 34 papers, 18 were excluded, and 16 were included in this systematic review [30,31,32,33,34,35,36,37,38,39,40,41,42,43,44,45].

### 3.2. Methodological Quality of Studies

All of the studies satisfied more than 70% of the proposed quality criteria of the JBI Critical Appraisal Tool [26], with an average quality score of 10 out of 13 (ranging from 9 to 12) (Table 2). Most of the studies had medium methodological quality [31,32,33,35,36,38,39,40,41,42,45], four studies had high quality [30,37,43,44] and one had excellent quality [34]. The two greatest areas of risk were performance bias (blinding of researchers and participants) and detection bias (blinding of outcome assessment). All included studies were evidence level 1.c—randomized controlled trials.

### 3.3. Study Characteristics

Table 3 presents the overall description of the included studies. All but two of the studies [39,43] were exclusively conducted in European countries, namely the Netherlands (n = 6), Germany (n = 4), Finland (n = 1), Portugal (n = 1), Spain (n = 1), and the United Kingdom (n = 1). The data collection periods occurred between 2003 [32] and 2022 [43], and the intervention period ranged from 2 weeks [32] to 12 months [30,36]. The sample sizes varied between 30 [40] and 432 [43] participants and included informal caregivers of adults with dementia (n = 12), disability or dependence for daily life activities (n = 2), cognitive impairment (n = 1), and cancer (n = 1).

### 3.4. Intervention Characteristics

The majority of the studies implemented a structured intervention programme compared to the provision of usual care (Table 3). Only one study evaluated an individualized programme in comparison with a regular programme and day care support [38]. One study evaluated an online training and support programme with an education-only e-book [42], while another compared training through the use of a tablet with the intervention app with the provision of a tablet without the intervention app.

All of the studies used structured questionnaires to gather quantitative data from the participants. The primary outcomes assessed mainly included characteristics related to the informal caregiver’s psychological well-being, namely quality of life (n = 12), perceived mental health (n = 11), burden (n = 10), sense of competence (n = 6), and self-efficacy (n = 6). Several different instruments were used, with only five instruments used by more than one study: the Zarit Burden Interview (n = 4), the Short-Form Health Survey (n = 3), the 20-item Centre for Epidemiological Studies Depression Scale (n = 3), the Short Sense of Competence Questionnaire (n = 6), and the Generalized Self-Efficacy Scale (n = 2). Overall, 27 outcomes were assessed using 46 different instruments.

### 3.5. Main Results of the Interventions

Although the majority of the studies described statistically significant and clinically relevant results for most of the outcomes assessed, five studies failed to describe any statistically significant association with the outcomes [30,39,41,44,45] (Table 3).

A synthesis of the associations for the health-related outcomes described for the person being cared for, the informal caregiver, or the health system, resulting from the implementation of programmes addressing informal caregivers’ needs, is presented in Table 4. Overall, studies reported a significant and direct association between the interventions implemented and QoL [33,34,42], mental health [31,35,38], self-efficacy [34,40,43], and coping strategies to address care [35]. Additionally, outcomes assessing psychological symptomatology (namely anxiety and depression) [31,35,37,42,43], burden [33,36,37], physical health symptomatology [35], and the caregiver–care relationship [32,33] were reported as significantly and inversely associated with the programmes addressing informal caregivers’ needs. Other outcomes, namely economic and social costs, quality-adjusted life years, and social support, did not show a significant association. Regarding patient outcomes, only one study reported a significant and positive effect on neuropsychiatric symptoms [38].

### 3.6. Type and Content of the Interventions

Table 5 describes the dimensions of the programmes aimed at meeting the needs of informal caregivers by the type of intervention. The programmes were analyzed and categorized according to six types of interventions: (1) caregiver monitoring, (2) management of older persons’ care, (3) training/education, (4) cognitive–behavioural strategies, (5) social support, and (6) physical activity and monitoring. Each type of intervention included different dimensions for a total of 24 dimensions. Interventions focusing on training and education of the caregivers (n = 14) and on cognitive–behavioural strategies (n = 7) were the most common across the studies, while interventions in the management of old persons’ care were found in only one study [34]. Information provision was reported in seven studies. Additionally, cognitive–behavioural strategies, namely coping with change, grief and loss, and stress management, were frequently reported across the studies, and were each included in 5 of the 16 studies. Regarding training and education strategies, the dimensions related to caregiver support and education, psychoeducation, and self-management were all described in four different interventions.

## 4. Discussion

The results of the current systematic review highlight the variety of interventions aimed at meeting the needs of informal caregivers, as well as the exploration of a wide range of dimensions across the programmes. Interventions including training, education, and cognitive–behavioural strategies were implemented in almost all studies, while strategies for the management of older persons’ care were implemented in only one study. Our work emphasizes the use of a wide range of health outcomes as the result of the interventions, with a particular focus on informal caregivers’ psychological well-being, which was evaluated through different instruments.

All studies implemented a structured and supervised programme to address informal caregivers’ needs. The results seem to support the achievement of positive health outcomes, particularly the improvement of mental health, quality of life, and emotional and physical well-being. Caring for loved ones requires a high level of skill, availability, and physical and emotional effort from informal caregivers [14,46,47]. These demands increase caregivers’ fatigue, stress, and burden, which may trigger harmful consequences for their health and impose a strain on healthcare systems [48,49,50]. Thus, it has been suggested that care services and programmes directed towards informal caregivers should adopt an interdisciplinary approach [51,52] to promote their health and well-being. However, no consensus exists on how to best prepare and support caregivers throughout the care trajectory [53,54]. Our results add to the current literature showing that interventions that focus on informal caregivers’ physical, psychological, social, and educational support contribute directly to better health outcomes. This awareness emphasizes the need to deeply understand the dynamic complexity of informal care and how to better prepare health and community settings to support both adults who are dependent for daily life activities and their informal caregivers.

None of the interventions reported adverse outcomes; however, five studies did not describe significant differences in the outcomes assessed after the implementation of the programmes [30,39,41]. The heterogeneous evaluation of the outcomes evaluated as well as the variability of the instruments used compromise the comparability of the results between the different studies and may partially explain the absence of significant results. In fact, the lack of specific and validated instruments to evaluate adults with dependence for daily life activities and informal caregivers may lead studies to neglect specific dimensions at the individual, institutional, and societal levels [55] that may not be addressed by instruments aimed at the general population.

Our study also supported the benefits of the implementation of programmes addressing informal caregivers’ needs for decreasing neuropsychiatric symptoms among people receiving care [38]. The authors reported a positive effect on behavioural and mood symptoms, possibly due to the development of personalized activities that matched participants’ preferences, abilities, and talents. These interventions had repercussions on the emotional impact of the symptoms on caregivers [38], supporting previous research demonstrating that psychosocial interventions for the person cared for and psychoeducational interventions for caregivers are beneficial for caregivers’ psychological well-being [56,57]. Thus, developing interventions with broader impacts that integrate psychosocial programmes tailored to the needs of the person who requires care and the informal caregiver in long-term care is crucial.

Recent studies have suggested that the provision of disease-specific and care-related information, training, and socioemotional support [15,17] may empower caregivers to provide quality care to the person in need of care, without detriment to their health, by increasing their knowledge and skills and helping them to cope with the negative consequences of caregiving [57,58,59]. Our results confirm that most of the programmes conducted in the last 12 years that aimed to meet the needs of informal caregivers included specific training, education, and cognitive–behavioural strategies, namely the provision of information and communication, training on the management of treatment and care, psychoeducation, stress management, and the development of coping skills and resources. However, few programmes [30,35] have focused on emotional and psychological support as a resource to improve caregivers’ psychological outcomes, although previous studies have stated that studies involving psychological interventions and including a psychologist as the operator yield significant improvements in depression, anxiety, well-being, and strain [52].

The results showed a lack of diversity in the studies’ countries of origin. This poor variability and limited cultural and ethnic sensitivity may influence the results [60] and hinder the real knowledge of the programmes that are available and implemented to address informal caregivers’ needs and, consequently, the development of inclusive policies and practises suitable for different populations. Furthermore, the short intervention and follow-up periods may preclude the assessment of long-term effects and maintenance of the results described. Twelve of the sixteen studies included informal caregivers of persons with dementia. These caregivers may have specific particularities given the progressive and often unpredictable nature of dementia [61], precluding the generalization of the results to all informal caregivers. Thus, investment in methodologically robust and sound cross-country and cross-cultural programmes designed to address the needs of informal caregivers of adults who are dependent for daily life activities in the short, medium, and long term should be considered.

Overall, the studies showed fair to high methodological quality with a higher risk of bias being related to blinding. These results were expected since the programmes assessed were nonpharmacological interventions, which increases the difficulty of blinding the participants to treatment assignment, the intervention providers, and the outcome evaluators, as previously reported [62]. However, the literature indicates that there are several ways in which the blinding of participants and key study personnel can be optimized that may help reduce the risk of performance and detection bias. For instance, participants can be blinded to hypotheses and to specific details of the intervention; those involved in outcome collection can be blinded to all elements except the outcome measures; investigators can be blinded to randomization and outcome measures in many instances; and statisticians can be blinded to most study elements [63]. Unfortunately, most of the included trials failed to discuss how intervention fidelity was maintained during of the trial, making it difficult to determine whether these methodological questions influenced the main findings [64]. Thus, future studies should be designed, developed, and evaluated carefully while taking into account the most recent reporting guidelines, to enhance scientific rigour.

### Strengths and Limitations

Despite the innovative nature of the current systematic review, some limitations must be acknowledged. First, the publication period of 12 years, from 2012 to 2024, was selected in response to the need to access the most up-to-date information on this topic. This period may have excluded some relevant papers published before 2012 from the current analysis. However, previous studies indicate that the conclusions of most systematic reviews are valid for approximately five years [65], and it has been suggested that systematic literature reviews should take into account evidence from the last five to ten years [66] to reflect the state of current knowledge in the field. Second, our search strategy was restricted to papers for which the full text was available, which may have limited our results. However, over the past decade, there has been a rise in the number of articles published in open-access journals, and some research funders are beginning to require their researchers to publish exclusively in open-access journals [67]. Thus, it is expected that the majority of relevant articles with high methodological quality published in this field, in the last decade, are available as full-text articles. Third, there was considerable methodological heterogeneity among the studies, particularly regarding the number of participants, the data collection period, and the duration of the interventions. Additionally, the variety of intervention programmes implemented and the diversity of the outcomes and the scales preclude the presentation of a meta-analysis. However, the selected databases, the search strategy, and the inclusion criteria were carefully structured and supported by the literature to capture the largest number and diversity of studies adequate for the objectives of this systematic review of the literature. Finally, the absence of studies conducted in countries other than Europe prevents the analysis of cross-country and cross-cultural studies, hindering the identification and integration of broader, more diverse, and more representative experiences and the comparison of findings.

## 5. Conclusions

The present systematic review adds to the growing body of evidence suggesting that programmes that address informal caregivers’ needs seem to contribute to better physical and psychological health outcomes through the promotion of caregivers’ educational support and the implementation of cognitive–behavioural strategies. This knowledge is essential for the design and implementation of person-centred care policies and practises, particularly with regard to the coproduction of health, shared governance, and care provision. These policies and practises contribute to empowering informal caregivers to provide quality care to adults who are dependent for daily life activities without detriment to their health. Framing informal care with an empowerment approach may contribute to addressing and minimizing the inadequacies and inequities of informal care.

Further research should clarify the main social and health gains for all stakeholders, including the person being cared for, the informal caregiver, and the health system, by implementing rigorous, broad, cross-cultural interventions tailored to informal caregivers’ physical, emotional, psychosocial, societal, and educational needs throughout the care trajectory.

## Figures and Tables

**Table 1:** Eligibility criteria according to the PICO(D) strategy [25].

PICOD		Inclusion Criteria	Exclusion Criteria
P	Population	(1) Informal caregivers defined as family members, friends, or any unpaid person providing or assisting patients without a background of formal medical education; (2) Patients aged 18 years old or above; (3) Informal caregivers of adults with dependence for daily life activities; (4) Community setting.	(1) Studies focusing on formal caregivers; (2) Studies focusing on children or adolescents; (3) Studies focusing on adults without dependence for daily life activities; (4) Institutional settings.
I	Intervention	(1) Programmess addressing informal caregivers needs to improve their experiences, health and well-being.	(1) Programmess not addressing informal caregivers’ needs.
C	Comparison	(1) Intervention with a control group receiving standard care, no intervention, or placebo.	(1) Intervention without a control group.
O	Outcome	(1) Health gains for the person being cared for, the informal caregiver, or the health system.	(1) Studies that did not report data on health gains.
D	Study Design	(1) Randomized controlled trial studies.	(1) Observational studies; (2) Nonoriginal studies (reviews, meta-analyses, study protocols, commentary, editorials, journal articles, conference proceedings and abstracts, reports, guidelines and grey literature and scale validations).

**Table 2:** Critical appraisal of the included studies.

Publication	JBI Critical Appraisal Checklist for Randomized Controlled Trials [26]														Evidence Level [27]
	1	2	3	4	5	6	7	8	9	10	11	12	13	Total	
Joling et al., 2013 [30]	1	1	1	0	0	1	1	1	1	1	1	1	1	11/13 (85%)	1.c
Rodriguez-Sanchez et al., 2013 [31]	1	1	1	0	0	0	1	1	1	1	1	1	1	10/13 (76%)	1.c
Ryynänen et al., 2013 [32]	1	0	1	0	0	0	1	1	1	1	1	1	1	9/13 (70%)	1.c
Berwig et al., 2017 [33]	1	1	1	0	0	0	1	1	1	1	1	1	1	10/13 (76%)	1.c
Boots et al., 2018 [34]	1	1	1	1	0	1	1	1	1	1	1	1	1	12/13 (92%)	1.c
Wilz et al., 2018 [35]	1	1	1	0	0	0	1	1	1	1	1	1	1	10/13 (76%)	1.c
Zwingmann et al., 2018 [36]	1	1	1	0	0	0	1	1	1	1	1	1	1	10/13 (76%)	1.c
Behrndt et al., 2019 [37]	1	1	1	1	0	0	1	1	1	1	1	1	1	11/13 (85%)	1.c
Dröes et al., 2019 [38]	1	1	1	0	0	0	1	1	1	1	1	1	1	10/13 (76%)	1.c
Gaugler et al., 2019 [39]	1	1	1	0	0	0	1	1	1	1	1	1	1	10/13 (76%)	1.c
Milders et al., 2019 [40]	1	1	1	0	0	0	1	1	1	1	1	1	1	10/13 (76%)	1.c
Schuit et al., 2022 [41]	1	0	1	0	0	0	1	1	1	1	1	1	1	9/13 (70%)	1.c
Teles et al., 2022 [42]	1	1	1	0	0	0	1	1	1	1	1	1	1	10/13 (76%)	1.c
Baker et al., 2023 [43]	1	1	1	0	0	1	1	1	1	1	1	1	1	11/13 (85%)	1.c
Beentjes et al., 2023 [44]	1	1	1	0	0	1	1	1	1	1	1	1	1	11/13 (85%)	1.c
Bielderman et al., 2024 [45]	1	1	1	0	0	0	1	1	1	1	1	1	1	10/13 (76%)	1.c
	100%	88%	100%	13%	0%	25%	100%	100%	100%	100%	100%	100%	100%		

0, No/Unclear; 1, Yes.

**Table 3:** Overall description of the studies included (n = 13).

Publication	Country	Period of Data Collection	Participants and Sample	Intervention	Outcomes (Instruments)	Main Results
Joling et al., 2013 [30]	The Netherlands	November 2007–November 2009 (12-month intervention period)	Family caregivers of patients with dementia (n = 192)	Family meeting intervention vs. Usual care	Caregiver and patient: − QoL (SF-12) ^1^ − Depression and anxiety (MINI) ^2^ − Quality-adjusted life years − Health system: − Economic costs (direct and indirect)	− No significant differences were found
Rodriguez-Sanchez et al., 2013 [31]	Spain	July 2008–November 2009 (3-week intervention period)	Family caregivers of relatives with dependence for daily life activities (n = 125)	Cognitive–behavioural treatment for managing dysfunctional thoughts about caregiving and training in self-help techniques vs. Usual care	Caregiver: − Self-perceived mental health (GHQ-12) ^3^ − Dysfunctional thoughts about caregiving (DAS) ^4^ − QoL (QoLQ) ^5^ − Burden (ZBI) ^6^	− Improvement in mental health (*p* = 0.01) − Decrease in dysfunctional thoughts about caregiving (*p* = 0.01)
Ryynänen et al., 2013 [32]	Finland	February 2003–April 2004 (2-week intervention period)	Caregivers of people with disability (n = 135)	Tailored multicomponent support intervention vs. Usual care	Caregiver: − Termination of the caregiver–care relationship − Caregiver’s depressive symptoms (SDS) ^7^ − QoL (15D scale) ^8^	− Lower termination of the caregiver–care relationship (*p* = 0.042)
Berwig et al., 2017 [33]	Germany	Not reported (6-month intervention period)	Informal caregivers of people with dementia (n = 92)	Individualized, psychoeducational, and skills-training evidence-based multicomponent intervention vs. Usual care	Caregiver: − Burden (ZBI) ^6^ − Mental health (PHQ-4) ^9^ − Somatization (PHQ -15) ^10^ − QoL (SF-12) ^1^ − Perceived social support (ESSI) ^11^ − Challenging behaviour (RMBPC-24) ^12^	− Decrease in burden (*p* = 0.017) − Improvement in QoL (*p* = 0.001) − Decrease in challenging behaviour frequency (*p* = 0.022) and reaction (*p* < 0.001)
Boots et al., 2018 [34]	The Netherlands	September 2014–December 2015 (8-week intervention period)	Family caregivers of community-dwelling people with mild dementia (n = 81)	Self-management partner in balance programme vs. Usual care	Caregiver: − Self-efficacy (CSES) ^13^ − Symptoms of depression (CES-D) ^14^ − Mastery (PMS) ^15^ − Anxiety (HADS-A) ^16^ − QoL (ICECAP-O) ^17^	− Increase in self-efficacy (*p* < 0.02) − Improvement in mastery (*p* = 0.01) − Improvement in QoL (*p* = 0.032)
Wilz, 2018 [35]	Germany	January 2012–November 2013 (6-month intervention period)	Family caregivers of people with dementia (n = 273)	Telephone-based cognitive–behavioural therapy vs. Usual care	Caregiver: − Depressive symptoms (CES-D) ^14^ − Physical health symptoms (GBB-24) ^18^ − Emotional well-being (Visual analogue scale) − Burden (Visual analogue scale) − Coping with burden and challenging behaviour (5-item scale)	− Fewer symptoms of depression (*p* = 0.043) − Fewer physical health symptoms (*p* = 0.019) − Improvement in emotional well-being (*p* = 0.001) − Improvement in coping with the care situation (*p* = 0.05) and the behaviour of the care recipient (*p* = 0.034)
Zwingmann et al., 2018 [36]	Germany	January 2012–December 2014 (12-month intervention period)	Caregivers of community-dwelling people with dementia (n = 317)	Dementia care management vs. Usual care	Caregiver: − Burden (BIZA-D) ^19^	− Decrease in caregiver burden (*p* < 0.005)
Behrndt et al., 2019 [37]	Germany	October 2014–March 2017 (6-month intervention period)	Informal caregivers of persons with cognitive impairment (n = 359)	Brief telephone intervention vs. Usual care	Caregiver: − Burden (BSFC-s) ^20^ − Well-being: level of depressiveness (WHO-5) ^21^ − Positive aspects of caregiving (BIZA-D) ^19^ − QoL (EQ-5D-5L) ^22^	− Decrease in subjective burden (*p* = 0.01) − Reduction in depressiveness (*p* < 0.01)
Dröes et al., 2019 [38]	The Netherlands	2016–2019 (6-month intervention period)	Informal caregivers of people with dementia (n = 282)	Individualized Meeting Centres Support Programme (iMCSP) vs. Regular MCSP and no day care support	Caregiver: − Sense of competence (SSCQ) ^27^ − Burden (NPI-Q—burden subscale) ^24^ − QoL (TOPICS-MDS) ^28^ − Happiness (TOPICS-MDS) ^28^ − Patient: − Self-esteem (RSE) ^23^ − Neuropsychiatric symptoms (NPI-Q) ^24^ − Experienced autonomy (EAS) ^25^ − QoL (DQoL) ^26^	− Positive effect on neuropsychiatric symptoms (*p* < 0.05) − Positive effect on caregiver happiness (*p* < 0.005)
Gaugler et al., 2019 [39]	USA	November 2017–June 2018 (6-month intervention period)	Family caregivers of persons living with Alzheimer’s disease or a related dementia (n = 132)	Remote activity monitoring (RAM) system vs. Usual care	Caregiver: − Self-efficacy and sense of competence (SSCQ) ^27^ − Distress (ZBI) ^6^ − Depressive symptoms (CES-D) ^14^ − Socioemotional support (5-item scale)	− No significant differences were found
Milders et al., 2019 [40]	UK	Not reported (3-month intervention period)	Informal caregivers of persons with dementia (n = 30)	Multicomponent nonpharmacological intervention vs. Usual care followed by delayed treatment	Caregiver: − Caregiver burden (ZBI) ^6^ − Caregiver QoL (SF-12) ^1^ − Sense of competence (SSCQ) ^27^ − Patient: − QoL (QoL-AD) ^29^ − Daily functioning (PSMS/IADL) ^30^ − Health system: − Health and social costs of the intervention	− Improvement in sense of competence (*p* < 0.05).
Schuit et al., 2022 [41]	The Netherlands	March 2019–August 2020 (3-month intervention period)	Partners of incurably ill cancer patients (n = 58)	eHealth self-management application Oncokompas vs. Usual care	Caregiver: − Burden (CSI+) ^31^ − Self-efficacy (GSE) ^32^ − QoL (EQ-5D) ^22^	− No significant differences were found
Teles et al., 2022 [42]	Portugal	March–May 2020 (6-month intervention period)	Informal caregivers of patients with dementia (n = 42)	Online training and support programme (iSupport-Portugal) vs. Education-only e-book	Caregiver: − Burden (ZBI) ^5^ − Depression and anxiety (HADS) ^16^ − Positive role appraisals (PAC) ^33^ − Self-efficacy (GSE) ^32^ − QoL (WHOQOL-BREF) ^34^	− Decrease in anxiety (*p* = 0.046) − Improvement in QoL (*p* = 0.029)
Baker et al., 2023 [43]	Australia, Germany, Norway, Poland and the UK	November 2019–July 2022 (12-week intervention period)	Cohabiting caregivers of persons with a diagnosis of dementia (n = 432)	Music intervention vs. Reading intervention vs. Usual care	Caregiver: − Emotional distress (NPI-Q distress score) ^24^ − Depression (PHQ-9) ^35^ − Resilience (RS14) ^36^ − Sense of competence (SSCQ) ^27^ − QoL (AQoL-6D) ^37^ − Quality of the caregiver and persons with dementia relationship (QCPR) ^38^ − Patient: − Neuropsychiatric symptoms (NPI-Q) ^24^ − Depression (MADRS) ^39^ − QoL (QoL-AD) ^29^ − Cognition (MMSE) ^40^	− Lower caregiver distress (*p* = 0.023) − Improvement in resilience (*p* = 0.011) − Decreasing QoL (*p* = 0.027)
Beentjes et al., 2023 [44]	The Netherlands	September 2018–January 2020 (3-month intervention period)	Informal caregivers of persons with mild cognitive impairment or dementia (n = 59)	Training to use a tablet and FindMyApps vs. Tablet without FindMyApps	Caregiver: − Sense of competence (SSCQ) ^27^ − Positive care experiences (PES) ^41^ − Patient: − Self-management (SMAS-S) ^42^ − Social participation (ASCOT ^43^; MSPP ^44^) − Self-efficacy (GSE) ^32^ − QoL (DQoL) ^26^	− No significant differences were found
Bielderman et al., 2024 [45]	The Netherlands	November 2016–March 2018 (18-week intervention period)	Family caregivers of people living with young-onset dementia (n = 60)	SPAN intervention vs. Usual care	Caregiver: − QoL (visual analogue scale; TOPICS-MDS ^28^) − Burden (visual analogue scale) − Emotional distress (NPI-Q distress score) ^24^ − Sense of competence (SSCQ) ^2^ − Patient: − Empowerment (SMAS-30) ^45^ − QoL (QOL-AD ^29^; EQ-5D-5L ^22^) − Neuropsychiatric symptoms (NPI-Q) ^24^ − Everyday disability (IDDD) ^46^ − Apathy (AES-10) ^47^	− No significant differences were found

QoL, quality of life; ^1^ Short-Form Health Survey; ^2^ Mini-International Neuropsychiatric Interview; ^3^ 12-Item General Health Questionnaire; ^4^ Dysfunctional Attitude Scale; ^5^ Quality of Life Questionnaire; ^6^ Zarit Burden Interview; ^7^ Zung’s Self-Rating Depression Scale; ^8^ 15D Instrument of Health-Related Quality of Life; ^9^ Patient Health Questionnaire—4 Items; ^10^ 9 Patient Health Questionnaire–Somatization Module; ^11^ Enriched Social Support Instrument; ^12^ Revised Memory and Behaviour Problem Checklist; ^13^ The Caregiver Self-Efficacy Scale; ^14^ 20-Item Centre for Epidemiological Studies Depression Scale; ^15^ 7-item Pearlin Mastery Scale; ^16^ Hospital Anxiety and Depression Scale; ^17^ Investigating Choice Experiments for the Preferences of Older People CAPability Measure for Older people; ^18^ Gießen Body Complaints List; ^19^ Berlin Inventory of Caregivers’ Burden with Dementia Patients; ^20^ Burden Scale for Family Caregivers Short; ^21^ WHO-5 Well-Being Index; ^22^ EuroQol Five Dimensions Questionnaire; ^23^ Rosenberg self-Esteem Scale; ^24^ Neuropsychiatric Inventory; ^25^ Experienced Autonomy Scale; ^26^ Dementia Quality of Life Scale; ^27^ Short Sense of Competence Questionnaire; ^28^ The Older Persons and Informal Caregivers Survey Minimum DataSet; ^29^ Quality of Life—Alzheimer’s Disease Scale; ^30^ Physical Self-Maintenance/Instrumental Activities of Daily Living; ^31^ Caregiver Strain Index+; ^32^ Generalized Self-efficacy Scale; ^33^ Positive Aspects of Caregiving; ^34^ World Health Organization Quality of Life—BREF; ^35^ Patient Health Questionnaire-9; ^36^ Resilience Scale-14; ^37^ Assessment of Quality of Life-6D Instrument; ^38^ Quality of Caregiver–Patient Relationship; ^39^ Montgomery Asberg Depression Rating Scale; ^40^ Mini Mental State Examination; ^41^ Positive Experience Scale; ^42^ Self-Management Ability Scale—Short Version; ^43^ Adult Social Care Outcomes Toolkit; ^44^ Maastricht Social Participation Profile; ^45^ Self-Management Ability Scale; ^46^ 20-item Interview for Deterioration in Daily Living Activities in Dementia; ^47^ 10-Item Apathy Evaluation Scale.

**Table 4:** Synthesis of the associations described for the health-related outcomes regarding the person being cared for, the informal caregiver, or the health system, after the implementation of the programmes addressing informal caregiver needs.

	Direct Association	Inverse Association	No Association
Caregiver			
Quality of life	✓✓✓ [33,34,42]	✓ [43]	✓✓✓✓✓✓✓✓ [30,31,32,37,38,40,41,45]
Psychological symptomatology ^1^		✓✓✓✓✓ [31,35,37,42,43]	✓✓✓✓✓✓ [30,32,33,34,39,45]
Mental health ^2^	✓✓✓ [31,35,38]		✓ [33]
Burden		✓✓✓ [33,36,37]	✓✓✓✓✓✓✓ [31,35,38,40,41,42,45]
Self-efficacy ^3^	✓✓✓ [34,40,43]		✓✓✓✓✓✓ [38,39,41,42,44,45]
Coping strategies ^4^	✓ [35]		✓✓ [37,44]
Physical health symptoms		✓ [35]	
Quality-adjusted life years			✓ [30]
Caregiver–care relationship ^5^		✓ [32,33]	✓ [43]
Socioemotional support			✓ [39]
Patient			
Quality of life			✓✓✓✓✓✓ [30,38,40,43,44,45]
Psychological well-being ^1^			✓✓✓✓ [30,40,43,45]
Quality-adjusted life years			✓ [30]
Neuropsychiatric symptoms	✓ [38]		✓✓ [43,45]
Self-efficacy ^6^			✓✓✓ [38,44,45]
Cognition			✓ [43]
Social participation			✓ [44]
Health system			
Costs ^7^			✓✓ [30,40]

^1^ Anxiety, depression; dysfunctional thoughts, somatization; emotional well-being, distress, apathy. ^2^ General mental health, emotional well-being, happiness. ^3^ Self-efficacy, mastery, sense of competence, resilience. ^4^ Coping with burden and challenging behaviour, positive care experiences. ^5^ Termination of the relationship, challenging behaviour, quality of relationship. ^6^ Self-esteem, autonomy, self-management, self-efficacy, empowerment. ^7^ Direct and indirect economic costs, health and social costs.

**Table 5:** Dimensions of the programmes aimed at the needs of informal caregivers by the type of intervention.

Type of Intervention	Dimensions
Caregiver monitoring	− Counselling/coaching [37,38] − Self-care [32,37,42]
Management of older persons’ care	− Acceptance [34] − Balance in activities [34] − Environment [34] − Communication [34]
Training/Education	− Information provision [33,38,40,41,43,44,45] − Communication and encouragement [40,42] − Management of treatment, care and activities [36,42,44,45] − Caregiver support and education [36,38,40,41] − Decision making [42] − Psychoeducation [30,31,33,35] − Self-management [31,35,37,41] − Physical and cognitive rehabilitation education [32,40,43]
Cognitive–behavioural strategies	− Problem-solving skills [30,33,35] − Stress management [33,34,35,37,40] − Changing dysfunctional cognition [33,34,35] − Coping with change, grief and loss [33,34,35,37,42] − Emotion regulation strategies [35]
Social support	− Family emotional and instrumental support [30] − Social relations and support [34] − Telephone support groups [33]
Physical activity and monitoring	− Monitoring of daily activity [39] − Caregiver physical fitness programme [32]

## Data Availability

Data will be available upon request (lmgp@uevora.pt).
